# Diagnostic Performance of the Hematology Analyzer XN-31 for Detecting *Plasmodium* Infections in School-Based Surveillance in Western Kenya

**DOI:** 10.4269/ajtmh.26-0027

**Published:** 2026-11-12

**Authors:** Wataru Kagaya, Takahiro Tougan, Bernard N. Kanoi, Ben-Yeddy Abel Chitama, Chim Chan, Protus Omondi, Brian Musyoka, Takatsugu Okai, James Kongere, Gordon Okomo, Jesse Gitaka, Akira Kaneko

**Affiliations:** ^1^ Department of Ecoepidemiology, Institute of Tropical Medicine (NEKKEN), Nagasaki University, Nagasaki, Japan;; ^2^ Department of Immunology, Graduate School of Biomedical Sciences, Nagasaki University, Nagasaki, Japan;; ^3^ Centre for Malaria Elimination, Institute of Tropical Medicine, Mount Kenya University, Thika, Kenya;; ^4^ Centre for Research in Infectious Diseases, Directorate of Research and Development, Mount Kenya University, Thika, Kenya;; ^5^ Malaria Laboratory, Graduate School of Medicine/Osaka International Research Center for Infectious Diseases, Osaka Metropolitan University, Osaka, Japan;; ^6^ Island Malaria Group, Department of Microbiology, Tumor and Cell Biology, Karolinska Institutet, Stockholm, Sweden;; ^7^ Center for Research in Tropical Medicine and Community Development, Nairobi, Kenya;; ^8^ Homa Bay County Government, Homa Bay, Kenya;; ^9^ Department of Protozoology, Institute of Tropical Medicine (NEKKEN), Nagasaki University, Nagasaki, Japan

## Abstract

Despite significant progress in malaria control, asymptomatic and submicroscopic infections remain important reservoirs sustaining transmission and are often undetected by conventional microscopy or rapid diagnostic tests (RDTs). The Automated Hematology Analyzer XN-31 (Sysmex, Kobe, Japan) has shown high diagnostic accuracy in clinical settings, but performance under active surveillance, particularly among asymptomatic individuals, has not been evaluated. In this study, we assessed XN-31 using samples from school-based active surveillance in western Kenya, where asymptomatic and submicroscopic infections are highly prevalent. Among 1,237 samples, 564 (45.6%) were positive for *Plasmodium* spp. by polymerase chain reaction (PCR), including 511 afebrile infections. Using PCR as the reference, XN-31 detected infections with a sensitivity of 70.2%, slightly higher than microscopy (65.1%), and a specificity of 98.4%, higher than RDT (79.8%) and similar to microscopy (98.7%). It detected 43 of 197 submicroscopic infections (21.8%), with a median parasite density of 57 parasites/*µ*L (interquartile range: 38–202). XN-31 identified 11 of 76 mixed infections of *Plasmodium falciparum* and non-*P. falciparum* compared with 5 by microscopy and 0 by RDT. Importantly, it also detected 8 of 14 *P. falciparum* infections suspected to carry *hrp2/3* deletions, which caused false-negative RDT results, comparable with microscopy. This is the first study to evaluate XN-31 for detecting asymptomatic infections, non-*P. falciparum* species, and *hrp2/3*-deleted parasites, which are key diagnostic challenges in endemic settings. Our findings support the potential of XN-31 as a practical and reliable surveillance tool that bridges between RDTs and PCR for enhanced parasite detection and accelerated malaria control efforts.

## INTRODUCTION

Malaria remains a major vector-borne parasitic disease, with *Plasmodium falciparum* being the deadliest species affecting humans. Despite extensive control efforts, malaria continues to impose a substantial burden, particularly in sub-Saharan Africa. In 2023, the WHO African Region accounted for 94% of the 263 million global malaria cases and 95% of the 597,000 malaria-related deaths as reported in the *World Malaria Report 2024*.^1^ One reason for its persistent transmission is the presence of asymptomatic infection. Older children and adults often develop partial immunity to malaria, reducing the likelihood of symptomatic infections. However, they can still carry the parasite without symptoms and at submicroscopic levels, and they serve as reservoirs that sustain malaria transmission.^2^^–^^5^ As these infections are asymptomatic, they typically do not seek diagnostic services and remain untreated. Consequently, actively identifying these infections with an appropriate tool is essential for accurately assessing transmission risks and enhancing the design of control strategies.

For many years, microscopic examination of Giemsa-stained blood films has been considered the gold standard for diagnosing malaria. However, the recognition of submicroscopic infections challenges this status, particularly in the realm of active surveillance. The use of rapid diagnostic tests (RDTs) for malaria screening has increased because of their simplicity, affordability, and ability to deliver results within 15 minutes. However, they do not provide quantitative results and may produce false positives owing to the persistence of circulating antigen after treatment.^6^ Furthermore, the emergence of *P. falciparum* strains lacking histidine-rich protein 2 (HRP2), which is the most common target for detection by *P. falciparum*-specific RDTs, has led to a rise in false-negative cases and diagnostic failures globally.^7^^,^^8^ In Kenya, studies have reported the presence of *P. falciparum* strains with *pfhrp2* and/or *pfhrp3* deletions among asymptomatic malaria infections, raising concerns about misdiagnosis and hidden reservoirs sustaining transmission.^9^^–^^11^ In addition, non-*P. falciparum* species, although less prevalent, can act as overlooked reservoirs of *Plasmodium* infection, posing additional diagnostic challenges as they are often underdetected because of the limited sensitivity of routine diagnostics. Molecular diagnostics, such as polymerase chain reaction (PCR), enable the detection of infections with higher sensitivity and specificity, although they require advanced equipment and expertise. Therefore, it is essential to develop innovative diagnostic methods that are both highly sensitive and operationally feasible to complement existing techniques and ensure effective active surveillance in malaria-endemic areas.

The Automated Hematology Analyzer XN-31 developed by Sysmex (Kobe, Japan) offers an alternative approach to rapidly quantify malaria-infected red blood cells using single-color flow cytometry and nucleic acid staining. Unlike PCR-based methods, the XN-31 can be operated by routinely trained laboratory staff with a smaller laboratory footprint equivalent to that of a conventional hematology analyzer. Although the cost per test is higher than that of RDT or microscopy, its lower personnel time and simplified workflow substantially offset this difference.^12^ A meta-analysis of previous studies reported a summary estimate of 97.6% sensitivity and 99.4% specificity against PCR, indicating excellent performance.^13^ However, these studies were hospital based and primarily focused on clinical cases, and none were conducted under active surveillance involving asymptomatic infections. Our previous work showed the high diagnostic accuracy of the XN-31 using blood samples after short-term storage and demonstrated its potential applicability for field surveillance.^14^

In this study, we assessed the performance of the XN-31 using samples collected from active school surveillance in the Lake Victoria region of Kenya, where we had previously identified a high prevalence of asymptomatic and submicroscopic infections.^15^ In addition, we examined its capability to detect *pfhrp2/3*-deleted *P. falciparum*, which had been demonstrated only in vitro,^16^ and non*-P. falciparum* infections, which are recognized as emerging threats.^17^^–^^20^ The findings of this study extend the applicability of this tool to active malaria surveillance and contribute to strengthening efforts to control the disease in endemic regions.

## MATERIALS AND METHODS

### Study design.

The primary objective of this study was to assess the performance of the XN-31 in diagnosing *Plasmodium* infections in the study population. The formula of Buderer^21^ was used for sample size estimation. Assuming a sensitivity of 80%, malaria prevalence of 10%, and absolute precision of 0.07, the required sample size was determined to be 1,254 participants.

A cross-sectional school survey was conducted in Suba South, Homa Bay County, Kenya from July 31 to August 6, 2023. This area was selected based on our previous surveillance (Kagaya W et al., unpblished data) to ensure an adequate number of participants. The region typically experiences a bimodal rainfall pattern, with a longer rainy season from March to July and a shorter rainy season from November to December. In the Lake Victoria basin, peak malaria transmission is observed 1 to 2 months after the rainy season.^15^ *Plasmodium falciparum* is the predominant malaria parasite, whereas *Plasmodium malariae* and *Plasmodium ovale* are less prevalent, and no *Plasmodium vivax* infections have been reported.^15^^,^^22^

Six primary schools in the area—Nyagwethe, Ngeri, Ragwe, Kisegi, Roo, and Kikubi—were selected based on accessibility and their ability to accommodate the survey schedule (Figure 1). An average of 210 students were recruited from each school and stratified by class. Consent forms detailing the study’s purpose, procedures, benefits, and risks were distributed 1 day before the survey. Parental or guardian consent was required for student participation, and verbal assent was obtained from each student upon enrollment. Demographic data (sex and age) and physical measurements (height and weight) were recorded, and axillary temperature was measured digitally. Hemoglobin level was measured with the HemoCue Hb 801 System (HemoCue AB, Ängelholm, Sweden). A 400-*µ*L sample of finger-pricked blood was collected for *Plasmodium* detection using RDT, the XN-31 analyzer, microscopy, and PCR as previously described.^14^

**Figure 1.**
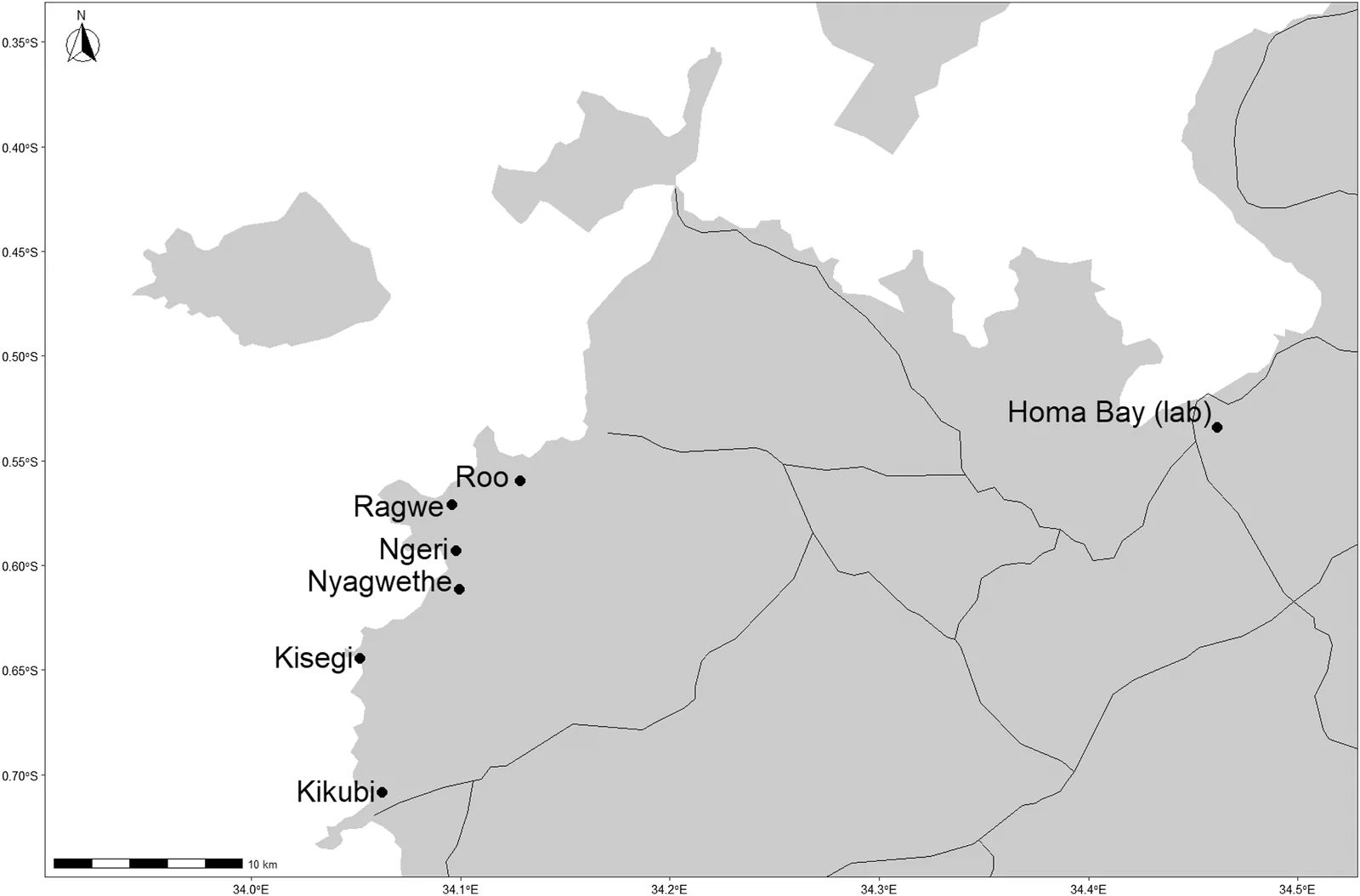
Map of the study sites and the laboratory where the analysis was conducted.

### Diagnostic methods.

#### Rapid diagnostic test for malaria.

*Plasmodium* infections were diagnosed on-site using Paracheck Pf RDT (Orchid Biomedical Systems, Goa, India) and Parascreen RDT (Zephyr Biomedicals, Verna, India) following the manufacturer’s instructions. The former detects the presence of PfHRP2 and is specific to *P. falciparum*, whereas the latter targets both PfHRP2 and the *Plasmodium* lactate dehydrogenase (pLDH) to detect *P. falciparum* and all human *Plasmodium* species, respectively. Participants who tested positive on any RDT received a standard course of artemether–lumefantrine following the dosage recommendations outlined in the Kenya Malaria Treatment Guidelines.^23^

#### Microscopy.

Thin and thick blood films were prepared on-site and transported to the laboratory in Mbita, where slide readings were performed. Thin film was fixed with methanol, and both films were stained with 3% Giemsa solution for 30 minutes. The stained blood films were independently examined by five experienced microscopists. Discrepant results were resolved by a third experienced microscopist who was blinded to the results of the first two examinations. The microscopists were certified by the WHO between 2021 and 2024 or the US Army Medical Research Unit—Kenya in 2018. A sample was declared negative after examination of at least 100 oil immersion microscopic fields at 1,000× magnification.^24^ Parasites were counted against 200 white blood cells (WBCs) or against 500 WBCs when fewer than 100 parasites were observed in 200 WBCs. Parasite density (parasites per microliter) was then calculated assuming a standard WBC count of 8,000/*µ*L. The parasite density was determined by taking the average of two readings.

#### XN-31 analyzer.

For detection with the XN-31, 250 *µ*L of capillary blood from the total sample was collected in a BD dipotassium ethylenediaminetetraacetic acid (K2-EDTA) Microtainer tube (BD, Franklin Lakes, NJ). After mixing by inversion, the tube was immediately stored at 4°C in a portable refrigerator (ESPEC MHD14F-D-ES, ESPEC, Osaka, Japan). Samples were transported daily to the main laboratory in the Homa Bay County Teaching and Referral Hospital and measured on the XN-31 (Figure 1). The time from sampling to measurement was 3 to 4 hours. Samples (60 *µ*L) were measured using the low-malaria mode optimized for detecting low densities of malaria-infected red blood cells. The detailed features of the system have been previously described,^25^ and the protocol was optimized based on our previous study.^14^ Positive samples are flagged as “Malaria?(P.f),” “Malaria?(others),” and/or “Malaria?(UNC),” corresponding to *P. falciparum* positive, non-*P. falciparum* malaria positive, and malaria positive with uncertain species, respectively. The differentiation of the first two flags is mainly based on the pattern of the scattergram.

#### Nested PCR for species diagnosis and quantitative PCR for quantification of P. falciparum.

Capillary blood (70 *µ*L) was spotted onto Whatman ET31 Chr filter paper (Whatman International, Maidstone, United Kingdom) and allowed to dry at ambient temperature. The dried blood spot (DBS) was placed in a small zipper plastic bag and stored at −20°C until DNA extraction. Dried blood spot samples were sent from Kenya to Japan in accordance with the Nagoya Protocol, and DNA extraction and PCR diagnosis were performed at Osaka Metropolitan University in Osaka, Japan. The QIAamp Blood Mini Kit (QIAGEN, Germantown, MD) was used to extract DNA from a quartered DBS (equivalent to 17.5 *µ*L of blood) according to the manufacturer’s instructions. DNA was eluted in 150 *µ*L of elution buffer (10 mM Tris–Cl and 0.5 mM EDTA, pH 9.0) and stored at −20°C. *Plasmodium* infections were detected by nested PCR targeting the multicopy mitochondrial cytochrome c oxidase subunit III (*cox3*) gene using 3 *µ*L of extracted DNA (equivalent to 0.35 *µ*L of blood) as a template.^26^ To quantify parasite density, quantitative polymerase chain reaction (qPCR) targeting the 18S ribosomal RNA gene was performed with technical triplicates on samples that tested positive for *P. falciparum* by nested PCR using the primers and probe that were previously described.^27^ Reactions were performed in a total reaction volume of 10 *µ*L using TaqMan Fast Advanced Master Mix for qPCR (Thermo Fisher Scientific Inc., Waltham, MA), and amplification was carried out on a QuantStudio 7 Flex Real-Time PCR System (Thermo Fisher Scientific Inc.) under the following cycling conditions: 95°C for 10 minutes followed by 40 cycles of 95°C for 15 seconds and 60°C for 60 seconds. Parasite density was estimated from a standard curve generated using serial dilutions of synthetized DNA with known plasmid copy numbers, and it is expressed as parasites per microliter of whole blood. Samples with cycle threshold (Ct) more than 35 were excluded from density-dependent analyses as undetermined.

#### Polymerase chain reaction for the detection of pfhrp2 and pfhrp3 deletion.

To investigate whether the XN-31 could detect parasites missed by RDT owing to *pfhrp2* and *pfhrp3* deletions, candidate samples were selected following our previous protocol.^11^^,^^28^ Briefly, all samples positive for *P. falciparum* by the *cox3* PCR but negative by both RDTs were tested with PCR amplifications of two single-copy genes: *P. falciparum* merozoite surface protein 1 (*pfmsp1*) and *P. falciparum* merozoite surface protein 2 (*pfmsp2*). Samples positive for both *pfmsp1* and *pfmsp2* were further evaluated for *pfhrp2* and *pfhrp3* deletions using nested PCR targeting exons 1 and 2 of *pfhrp2* and *pfhrp3*. Because the nonexpression of PfHRP2 and/or PfHRP3 proteins arises from frameshift mutations caused by exon 1 and exon 2 deletions (regions corresponding to the target epitope of PfHRP2-based RDT), samples failing to amplify either or both loci were considered to harbor *P. falciparum* with *pfhrp2* and/or *pfhrp3* deletions.

## STATISTICAL ANALYSES

Febrile cases were defined as an axillary temperature greater than or equal to 37.5°C. Anemia status was classified as nonanemia, mild, moderate, or severe according to age- and sex-specific thresholds defined by the WHO.^29^ Using PCR as the reference, the diagnostic performances of microscopy, RDT, and the XN-31 were evaluated. First, overall performance was determined based on binarized (i.e., positive/negative) test results regardless of *Plasmodium* species composition. For RDT, any positive result by Paracheck Pf RDT or Parascreen RDT was considered positive. For the XN-31, all samples with one or more of these three flags (“Malaria?(P.f),” “Malaria?(others),” and/or “Malaria?(UNC)”) were counted as positive, and an abnormal scattergram was counted as negative unless indicated otherwise. Second, samples were categorized by fever and anemia status, and the performance of the diagnostic tests was compared between subgroups. Third, the relationship between parasite density and diagnostic sensitivity was evaluated among PCR-positive infections with quantifiable parasite density by qPCR. Infections were stratified into two density categories (<200 and >200 parasites/*µ*L), and sensitivity with 95% CIs was calculated for each method within each category. In addition, sensitivity was modeled as a continuous function of log_10_-transformed qPCR parasite density using logistic regression for each diagnostic method. Lastly, the performance in *P. falciparum* and non-*P. falciparum* species identification was separately evaluated. Because the Parascreen RDT cannot distinguish *P. falciparum* and mixed *Plasmodium* infections when both PfHRP2 and pLDH were detectable in blood samples, the number of non-*P. falciparum* infections could not be ascertained. Rapid diagnostic test was thus excluded in the comparison of non-*P. falciparum* diagnostic performance.

Performance indicators, including sensitivity, specificity, positive predictive value, and negative predictive value, were calculated. The relative diagnostic sensitivity between fever and anemia subgroups was calculated using univariable robust Poisson regression and expressed as a sensitivity ratio.^30^ The comparisons of parasite counts between XN-31 and microscopy and between XN-31 and qPCR were evaluated using a Bland–Altman plot. All analyses were performed using R v. 4.5.1 (R Foundation, Vienna, Austria).

## RESULTS

### Participant characteristics.

Of 1,320 enrolled participants, 1,237 were included in the analysis after excluding those with incomplete data. The demographic characteristics of the participants are presented in Table 1. Among them, 8% (*n* = 99) were febrile (≥37.5°C). The prevalence of anemia was 22.4% (*n* = 277), with 11 individuals classified as severely anemic.

**Table 1:** Description of the study participants

Characteristics	All (*N* = 1,237)
Age, median (IQR)	11 (8–13)
Sex, male, *n* (%)	576 (46.6)
Fever, *n* (%)	99 (8.0)
Anemia, *n* (%)	
No	960 (77.6)
Mild	131 (10.6)
Moderate	135 (10.9)
Severe	11 (0.9)
Net use, *n* (%)	656 (53.0)

IQR = interquartile range.

### Diagnostic sensitivity and specificity.

For the detection of any *Plasmodium* species, the positivity rates were as follows: 45.6% (*n* = 564) by PCR, 30.4% (*n* = 376) by microscopy, 50.9% (*n* = 630) by RDT, and 32.9% (*n* = 407) by XN-31. The species composition of the 564 PCR-positive cases was as follows: 471 *P. falciparum*, 10 *P. ovale*, 7 *P. malariae*, 34 *P. falciparum* and *P. ovale* coinfections, 29 *P. falciparum* and *P. malariae* coinfections, and 13 triple infections (*P. falciparum*, *P. ovale*, and *P. malariae*). Among 547 *P. falciparum*-positive cases, 360 (65.8%) were successfully amplified by qPCR, and the median parasite density was 378 parasites/*µ*L (interquartile range [IQR]: 145–1,581) (Supplemental Figure 1A). The numbers of infections detected by the two RDTs are presented in Supplemental Table 1. Using PCR as the reference, the overall sensitivity values for microscopy, RDT, and XN-31 were 65.1% (95% CI: 61.0%–68.9%), 87.6% (95% CI: 84.6%–90.1%), and 70.2% (95% CI: 66.3%–73.8%), respectively. Overall specificity values were 98.7% (95% CI: 97.5%–99.3%) for microscopy and 98.4% (95% CI: 97.1%–99.1%) for XN-31, but they were lower for RDT at 79.8% (95% CI: 76.6%–82.7%) (Table 2, overall).

**Table 2:** Diagnostic performance of PCR, microscopy, RDT, and XN-31 with overall population, afebrile and febrile populations, and non-anemic and anemic populations

Test	Positive, *n* (%)	TP	FP	FN	Sensitivity, % (95% CI)	Specificity, % (95% CI)	PPV, % (95% CI)	NPV, % (95% CI)	Relative Sensitivity (95% CI)
Overall									
PCR	564 (45.6)								
Microscopy	376 (30.4)	367	9	197	65.1 (61.0–68.9)	98.7 (97.5–99.3)	97.6 (95.5–98.7)	77.1 (74.2–79.8)	
RDT	630 (50.9)	494	136	70	87.6 (84.6–90.1)	79.8 (76.6–82.7)	78.4 (75.0–81.4)	88.5 (85.7–90.8)	
XN	407 (32.9)	396	11	168	70.2 (66.3–73.8)	98.4 (97.1–99.1)	97.3 (95.2–98.5)	79.8 (76.9–82.4)	
Afebrile									
PCR	511 (44.9)								
Microscopy	332 (29.2)	324	8	187	63.4 (59.1–67.5)	98.7 (97.5–99.4)	97.6 (95.3–98.8)	76.8 (73.8–79.6)	0.78 (0.68–0.9)
RDT	570 (50.1)	443	127	68	86.7 (83.5–89.4)	79.7 (76.4–82.7)	77.7 (74.1–80.9)	88.0 (85.1–90.4)	0.9 (0.85–0.96)
XN	362 (31.8)	352	10	159	68.9 (64.7–72.7)	98.4 (97.1–99.1)	97.2 (95.0–98.5)	79.5 (76.5–82.2)	0.83 (0.72–0.95)
Febrile									
PCR	53 (53.5)								
Microscopy	44 (44.4)	43	1	10	81.1 (68.6–89.4)	97.8 (88.7–99.6)	97.7 (88.2–99.6)	81.8 (69.7–89.8)	
RDT	60 (60.6)	51	9	2	96.2 (87.2–99.0)	80.4 (66.8–89.3)	85.0 (73.9–91.9)	94.9 (83.1–98.6)	
XN	45 (45.5)	44	1	9	83.0 (70.8–90.8)	97.8 (88.7–99.6)	97.8 (88.4–99.6)	83.3 (71.3–91.0)	
Nonanemic									
PCR	390 (40.6)								
Microscopy	259 (27.0)	253	6	137	64.9 (59.9–69.6)	98.9 (97.7–99.6)	97.7 (95.0–99.1)	80.5 (77.3–83.3)	0.99 (0.87–1.13)
RDT	427 (44.5)	333	94	57	85.4 (81.5–88.7)	83.5 (80.2–86.5)	78.0 (73.8–81.8)	89.3 (86.4–91.8)	0.92 (0.87–0.98)
XN	277 (28.9)	269	8	121	69.0 (64.1–73.5)	98.6 (97.3–99.4)	97.1 (94.4–98.7)	82.3 (79.2–85.1)	0.95 (0.84–1.06)
Anemic									
PCR	174 (62.8)								
Microscopy	117 (42.2)	114	3	60	65.5 (57.9–72.5)	97.1 (91.7–99.4)	97.4 (92.7–99.5)	62.5 (54.5–70.0)	
RDT	203 (73.3)	161	42	13	92.5 (87.6–96.0)	59.2 (49.1–68.8)	79.3 (73.1–84.7)	82.4 (71.8–90.3)	
XN	130 (46.9)	127	3	47	73.0 (65.7–79.4)	97.1 (91.7–99.4)	97.7 (93.4–99.5)	68.0 (59.8–75.5)	

FN = false negative; FP = false positive; NPV = negative predictive value; PCR = polymerase chain reaction; PPV = positive predictive value; RDT = rapid diagnostic test; TP = true positive.

The relative sensitivities of microscopy, RDT, and XN-31 across various statuses are presented in Table 2. All three diagnostics exhibited lower sensitivity in the afebrile group compared with the febrile group. The relative sensitivity, which was calculated using robust Poisson regression, was 0.78 (0.68–0.9) for microscopy, 0.90 (0.85–0.96) for RDT, and 0.83 (0.72–0.95) for XN-31 (Table 2, febrile and afebrile). The median parasite density by qPCR was 353 parasites/*µ*L (IQR: 138–1,220) in afebrile participants (*n* = 319) and 1,888 parasites/*µ*L (IQR: 238–14,000) in febrile participants (*n* = 41) (Supplemental Figure 1B). Between nonanemic and anemic groups, the relative sensitivity was similar for the three diagnostics: 0.99 (0.87–1.13) for microscopy, 0.92 (0.87–0.98) for RDT, and 0.95 (0.84–1.06) for XN-31 (Table 2, nonanemic and anemic). The median parasite density by qPCR was 326 parasites/*µ*L (IQR: 116–1,230) in nonanemic participants (*n* = 246) and 594 parasites/*µ*L (IQR: 204–2,090) in anemic participants (*n* = 114) (Supplemental Figure 1C). Rapid diagnostic tests had notably low specificity across all subgroups (Table 2).

Among 564 infections confirmed by PCR, 197 (34.9%) were undetected by microscopy and thus, considered as submicroscopic. Of these, RDTs identified 141 infections with 71.6% (64.9%–77.4%) sensitivity, whereas the XN-31 detected 43 infections with 21.8% (16.6%–28.1%) sensitivity. Among the 43 submicroscopic infections detected by the XN-31, the median parasite density was 57 parasites/*µ*L (IQR: 38–202) by the XN-31 and 58 parasites/*µ*L (IQR: 28–118) by qPCR.

When stratified by parasite density using a cutoff of 200 parasites/*µ*L by qPCR, the sensitivity of microscopy declined from 96.7% (93.7%–98.3%; *n* = 246) at higher densities to 75.4% (66.8%–82.4%; *n* = 114) at low densities. In contrast, RDT and XN-31 sensitivities showed only modest declines: from 99.6% (97.7%–99.9%) to 94.7% (89.0%–97.6%) for RDT and from 99.2% (97.1%–99.8%) to 91.2% (84.6%–95.2%) for XN-31. These density-stratified findings were consistent with logistic regression models describing sensitivity as a continuous function of qPCR-quantified parasite density for each diagnostic method (Figure 2).

**Figure 2.**
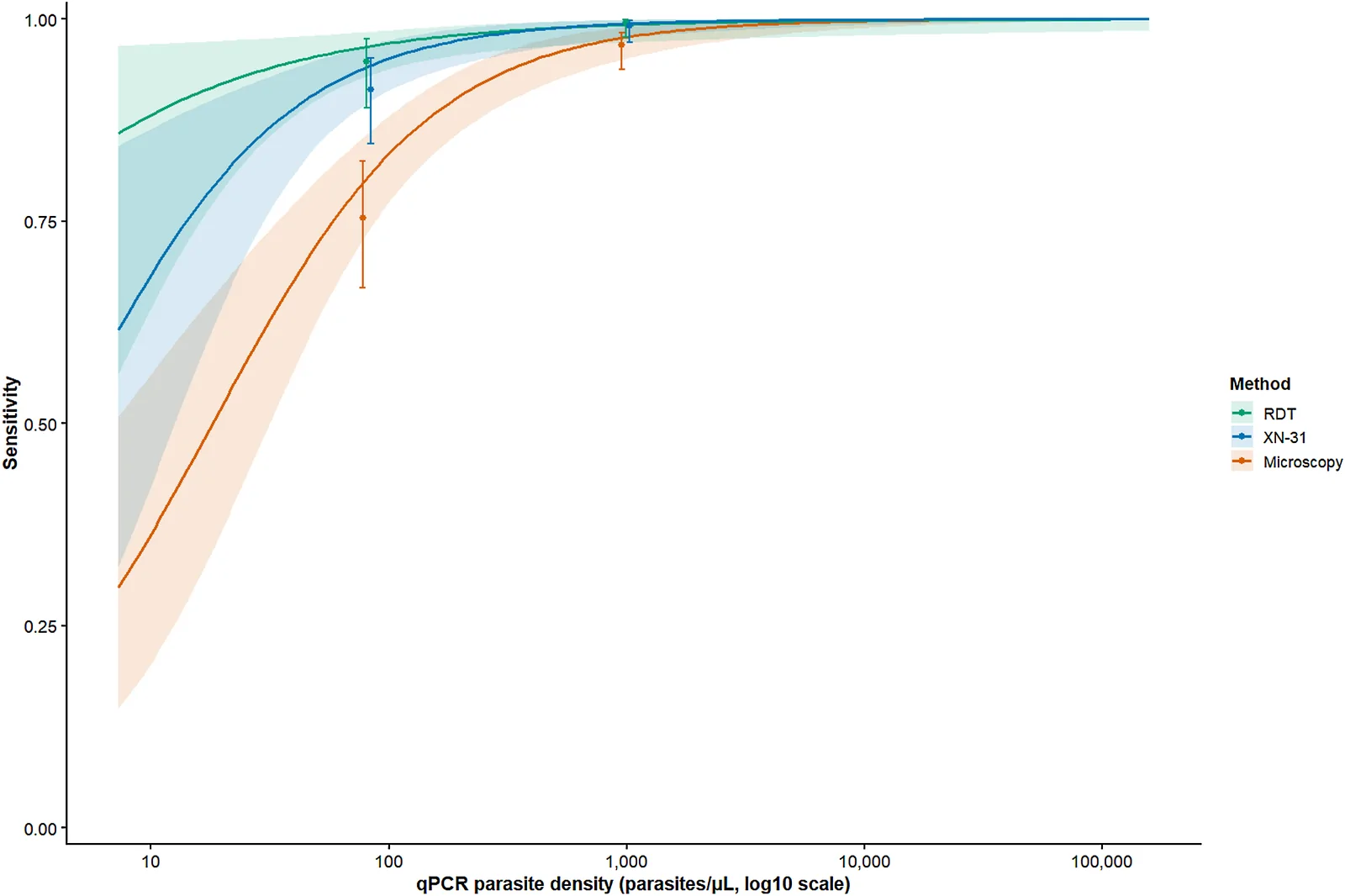
Density-dependent sensitivity of microscopy, rapid diagnostic test (RDT), and XN-31. Solid lines represent logistic regression fits for each diagnostic method, with shaded bands indicating 95% CIs. Points represent sensitivity estimates stratified by parasite density category (<200 and >200 parasites/*µ*L) plotted at the median parasite density within each category. qPCR = quantitative polymerase chain reaction.

### Comparison of parasite counts by the XN-31, microscopy, and qPCR.

Among the 361 *Plasmodium*-positive samples detected by both microscopy and the XN-31, the median parasite density determined by microscopy was 587 parasites/*µ*L (IQR: 227–2,427), and the median density measured by the XN-31 was 997 parasites/*µ*L (IQR: 355–4,147). Parasite counts obtained using the XN-31 and microscopy were compared using a Bland–Altman plot (Figure 3A). Microscopy yielded parasite density estimates that were, on average, 0.65 times those obtained by the XN-31. The limits of agreement ranged from 0.21-fold to 1.98-fold. The plot did not indicate a significant trend in the differences across the spectrum of parasite densities, suggesting minimal proportional bias.

**Figure 3.**
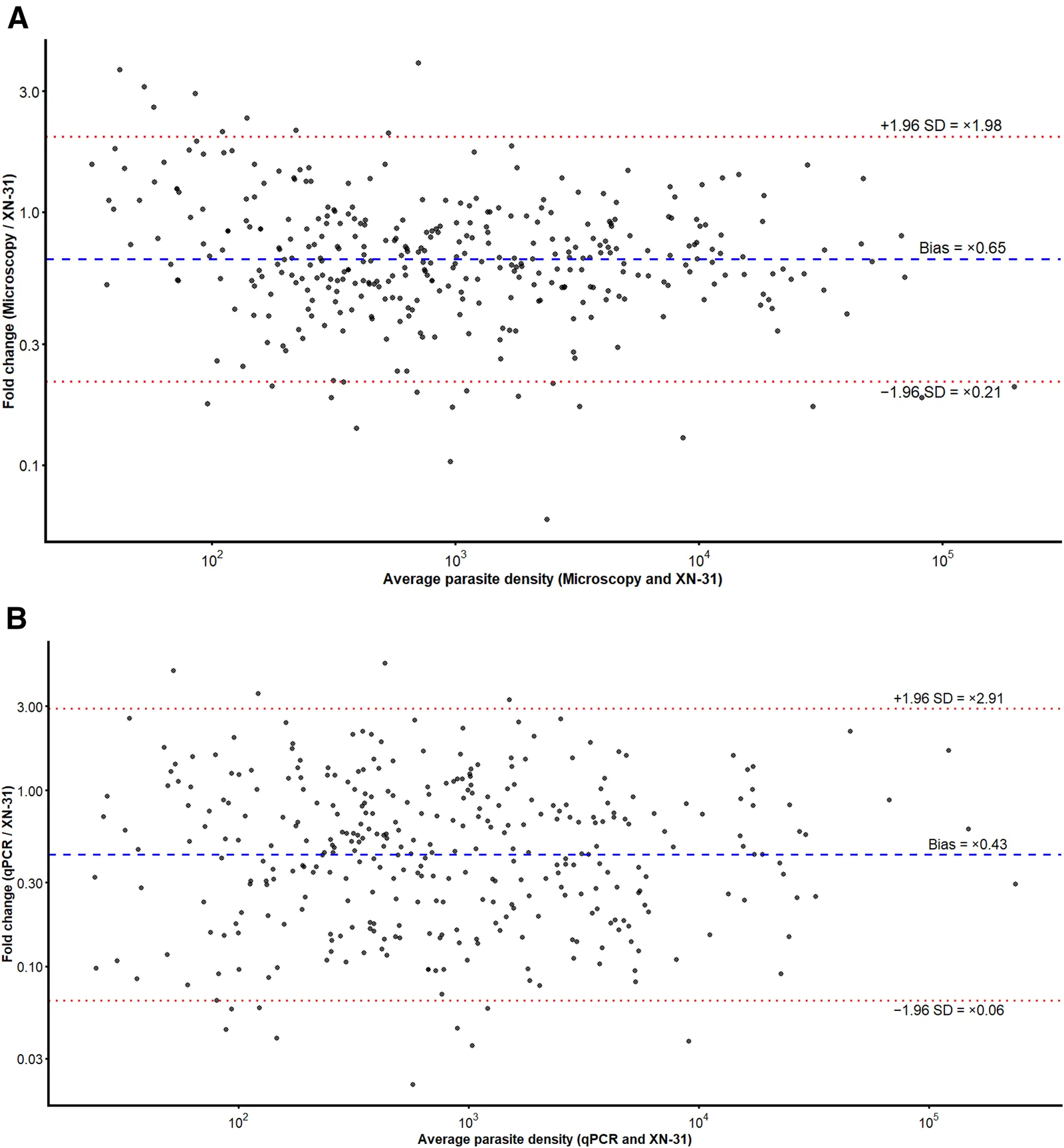
Bland–Altman plots to compare the parasite counts measured by (**A**) microscopy and XN-31 and (**B**) quantitative polymerase chain reaction (qPCR) and XN-31.

For the 348 *P. falciparum*-positive samples detected by both qPCR and the XN-31, the median parasite density was 407 parasites/*µ*L (IQR: 154–1,648) by qPCR and 989 parasites/*µ*L (IQR: 352–4,014) by the XN-31. The Bland–Altman plots for qPCR and XN-31 (Figure 3B) showed that qPCR estimates were on average 0.43-fold lower than XN-31 estimates, with limits of agreement ranging from 0.06-fold to 2.91-fold. No trend suggesting proportional bias was observed.

### Detection of *P. falciparum* and non-*P. falciparum* species.

All *Plasmodium* infections detected by PCR were classified as *P. falciparum* single infections (Pf), non-*P. falciparum* single infections (others), or mixed infections of *P. falciparum* and non-*P. falciparum* (Pf + others) (Table 3). Among samples infected solely with *P. falciparum* (*n* = 471), microscopy, RDT, and the XN-31 identified 291 (61.8%), 267 (56.7%), and 193 cases (41.0%), respectively. Among samples in which the XN-31 detected *P. falciparum*, the median parasite density was 569 parasites/*µ*L (IQR: 205–2,262) in samples with a Pf-only flag and 617 parasites/*µ*L (IQR: 225–1,892) in samples with a concurrent others flag. Given that the RDT targeting pLDH also targets *P. falciparum*, 151 cases classified as Pf + PAN can be interpreted as single infections of *P. falciparum*, indicating that RDTs demonstrate the highest sensitivity (88.7%), albeit with a lower specificity of 60.1% compared with microscopy (80.4%) and the XN-31 (79.8%). Overall, the trend mirrored the performance of all *Plasmodium* spp.

**Table 3:** Diagnostic performance of microscopy, RDT, and XN-31 for PCR-confirmed *P. falciparum* (Pf) and non-*P. falciparum* species (Others)

Diagnosis	PCR				Total
	Pf	Others	Pf + Others	Negative	
Microscopy					
Pf	291	1	61	9	362
Others	0	4	2	2	8
Pf + others	2	2	5	0	9
Negative	178	10	8	662	858
RDT					
Pf	267	5	40	132	444
PAN	1	0	0	1	2
Pf + PAN	151	0	30	3	184
Negative	52	12	6	537	607
XN					
Pf	193	0	46	3	242
Others	17	2	6	3	28
Pf + others	56	2	11	1	70
UNC	55	3	5	4	67
Abn_sc	25	2	2	60	89
Negative	125	8	6	602	741
Total	471	17	76	673	1,237

Abn_sc = abnormal scattergram; PAN = pan-*Plasmodium* infection; PCR = polymerase chain reaction; Pf = *Plasmodium falciparum* single infection; RDT = rapid diagnostic test; UNC = malaria positive with uncertain species.

Regarding single infections of other species (*n* = 17), microscopy and the XN-31 detected four and two samples, respectively, whereas RDT (pan-*Plasmodium* diagnosis with Parascreen RDT) failed to detect any samples. In cases of mixed infections of *P. falciparum* and non-*P. falciparum* (*n* = 76), microscopy detected 5 samples, and the XN-31 identified 11 samples as mixed infections (flagged with both “Malaria?(P.f)” and “Malaria?(others)”). Thirty samples tested positive with both the *P. falciparum*-specific RDT and the pan-*Plasmodium* RDT; however, these can still be interpreted as single infections of *P. falciparum*. Parasite density did not differ significantly across the three XN-31 flag patterns among these mixed infections (*P* = 0.50, Kruskal–Wallis test): 385 parasites/*µ*L (IQR: 296–1,061) with both flags (*n* = 10), 402 parasites/*µ*L (IQR: 205–1,294) with a Pf-only flag (*n* = 44), and 292 parasites/*µ*L (IQR: 146–373) with an others-only flag (*n* = 5).

### Detection of HRP2-deleted *P. falciparum*.

A total of 65 *cox3* PCR-positive *P. falciparum* samples were negative by both the *P. falciparum*-diagnosis with Parascreen RDT and Paracheck Pf RDT, and they were suspected to harbor *P. falciparum* with *pfhrp2/3* deletions. Among these samples, 17 were successfully amplified using single-copy genes *pfmsp1* and *pfmsp2*. Of these 17 samples, 14 exhibited no amplification of either *pfhrp2* or *pfhrp3*. Of these 14 samples with *pfhrp2/3* deletions, the XN-31 detected eight infections, whereas microscopy detected seven; six infections were detected by both the XN-31 and microscopy. Additionally, 2 of 14 samples displayed abnormal scattergrams on the XN-31. The median parasite density determined by the XN-31 was 329.5 parasites/*µ*L (IQR: 138–449.7) compared with 280 parasites/*µ*L (IQR: 173.3–386.7) by microscopy.

## DISCUSSION

The diagnosis of malaria has traditionally relied on microscopy and RDT, each with distinct advantages and limitations. Depending on the specific context and objectives, it is essential to use different diagnostic methods, underscoring the importance of having multiple diagnostic options available. The Sysmex XN-31 can identify *P. falciparum* and other *Plasmodium* species individually and deliver parasite counts within 1 minute. In our previous study, we demonstrated the capability of the XN-31 to diagnose malaria using capillary blood samples after 24 hours of storage at 2°C to 8°C, suggesting its applicability for active surveillance in remote settings,^14^ although a stable supply of electricity is required for its operation. In the current study, we used blood samples collected from a school-based mass survey to compare the diagnostic performance of the XN-31 with conventional methods: namely, microscopy, RDT, and PCR. To our knowledge, this is the first study to include asymptomatic *Plasmodium* infections, non-*P. falciparum* species, and *P. falciparum* with *pfhrp*2/3 deletions in the evaluation of XN-31 diagnostic performance and to demonstrate its ability to detect these diagnostically challenging infections. Additionally, this is the first study to evaluate the performance of XN-31 in a density-dependent manner. Although one previous study compared the XN-31 with qPCR,^25^ it did not examine the density dependency of diagnostic performance. Together, these findings support the potential use of the XN-31 in diverse contexts, including mass screening surveillance.

Previous meta-analyses have reported that the sensitivity and specificity of the Sysmex hematology analyzer using PCR as the standard were 97.6% (95% CI: 83.2–99.7) and 99.4% (95% CI: 98.5–99.8), respectively.^13^ Our findings on specificity (98.4%; 95% CI: 97.1–99.1) align with these results; however, the sensitivity observed in our study was lower at 70.2% (95% CI: 66.3–73.8). This discrepancy is likely attributable to the fact that our study was conducted as part of active surveillance, which included a significant number of asymptomatic and/or submicroscopic infections. Notably, most studies included in the meta-analysis focused on suspected malaria cases, or in instances where asymptomatic cases were targeted, the parasite density was relatively higher than in our study, where the median parasite density by microscopy was 587 parasites/*µ*L (IQR: 227–2,427). Upon evaluating the sensitivity of the XN-31 in comparison with microscopy, it can be concluded that the XN-31 exhibits superior performance. Specifically, the XN-31 identified 43 of 197 submicroscopic infections with a median parasite density of 57 parasites/*µ*L (IQR: 38–202), thereby demonstrating its ability to detect low-density malaria infections. Consistent with this, the density-dependent sensitivity analysis confirmed that the XN-31 outperformed microscopy particularly at low parasite densities (<200 parasites/*µ*L), close to the conventional limit of detection by microscopy.

Low RDT specificity in this study reflected the large number of false positives (Table 2). A high degree of agreement between the Paracheck Pf RDT and Parascreen RDT (Supplemental Table 1) suggested a common cause for false positivity. A likely explanation is the detection of residual PfHRP2, which may persist in the bloodstream for up to 36 days postclearance.^6^ This phenomenon varies seasonally and has been shown to be more common during high-transmission periods in Uganda and Burkina Faso.^31^^,^^32^ Our survey was conducted at the conclusion of the long rainy season in August when the numbers of current and recent infections were expectedly high. Another potential factor contributing to false positives in RDT is observer bias. The interpretation of the RDT results is based on the visibility of the test band(s). Several studies have documented false-positive bands when the RDT was examined beyond the manufacturer’s recommended development time (typically 15–20 minutes).^33^^,^^34^ Because of the large number of participants enrolled in a short allocated time frame, delayed reading of some RDTs may have occurred, potentially inflating the false-positive rate. The XN-31 directly detects malaria-infected red blood cells through an automated process and therefore, is not affected by residual antigens or observer-dependent variability. This is particularly relevant for posttreatment monitoring, and the concept has been validated in other settings.^35^

We further compared the diagnostic performance by fever and anemia status. When comparing between the afebrile and febrile groups, all three tests exhibited a relative sensitivity below one, indicating that these tests performed better in identifying *Plasmodium* infections in febrile individuals than in afebrile individuals. This finding is consistent with the fact that febrile or symptomatic infections typically present with higher parasitemia, resulting in increased sensitivity. Nevertheless, even among afebrile populations, both microscopy and the XN-31 demonstrated sensitivities exceeding 60%. Notably, the XN-31 consistently exhibited superior sensitivity compared with microscopy and given its specificity, may be preferable to RDT. These results support the use of the XN-31 in mass screening, where many infections are afebrile or asymptomatic. Meanwhile, subgroup analysis by anemia status revealed no substantial differences in the sensitivity or specificity of the three diagnostic tests. This likely reflects the diverse etiologies of anemia in malaria-endemic settings,^36^ resulting in study populations that are broadly comparable with respect to malaria infection status. Consequently, stratification by anemia did not significantly influence the test performance. One limitation is that because the study relied on school-based active surveillance, children in poor health may have been absent and thus, excluded from the sample. Nevertheless, these findings provide important insights into the utility of active surveillance.

A key advantage of the XN-31 is its ability to provide parasite counts, a surrogate marker of infection severity. When the parasite counts obtained by the XN-31 were compared with those obtained by microscopy and qPCR, the results were generally consistent. This study is the first to demonstrate concordance between the XN-31 and qPCR and that between the XN-31 and microscopy in low-density infection cases. Microscopy and qPCR showed a slight fixed bias at lower counts (factors of 0.65 and 0.43, respectively); however, the limits of agreement included the line of no bias. Regarding proportional bias, no significant evidence was observed. A minor trend suggested that higher parasitemia yielded higher counts in XN-31, but this did not affect the overall agreement.

Recent reports have indicated an increase in non-*P. falciparum* malaria infections, which has heightened attention on these species, specifically *P. malariae* and *P. ovale*.^17^^–^^20^ Some studies have even documented severe cases of these infections, which continue to pose significant threats to travelers and infants with limited immunity.^37^^,^^38^ Although artemisinin combination therapy is used for these infections, *P. ovale* requires attention because of hypnozoites, and *P. malariae* is also frequently associated with prolonged recurrence.^39^^,^^40^ Thus, species differentiation remains an important component of malaria diagnosis. The XN-31 device is engineered to provide three distinct indicators pertinent to species differentiation: “Malaria?(P.f),” “Malaria?(others),” and “Malaria?(UNC).” These diagnostic indicators are primarily based on the size of the infected red blood cells, the presence of the trophozoite stage in the bloodstream, and the number of ring-stage parasites within a single red blood cell as detected using flow cytometry techniques.^41^ We further evaluated the device performance in mixed infections of *P. falciparum* and non-*P. falciparum* species. As shown in Table 3, the overall species-specific concordance of the XN-31 with PCR was lower than that of microscopy and RDT. Notably, however, the XN-31 identified more mixed infections than microscopy (11 of 76 cases versus 5 of 76 cases, respectively), suggesting some capacity to detect low-density non-*P. falciparum* coinfections that microscopy readily misses. In most instances of mixed infections, non-*P. falciparum* species often exhibit low parasitemia and can be obscured by the predominance of *P. falciparum*, although in our data, qPCR-quantified parasite density did not differ significantly across XN-31 flag categories, suggesting that factors other than parasite density may also contribute to this misclassification. Given this, the clinical utility of the XN-31’s species-specific flags warrants further evaluation with larger sample sizes. In the case of RDT, even when results are positive for both *P. falciparum*-specific antigen and *Plasmodium* universal antigen, it remains impossible to distinguish between *P. falciparum* single and mixed infections.

In addition to the three previously mentioned flags, the XN-31 also provides feedback labeled as “Abn_sc,” which indicates an abnormal scattergram. The cause remains unclear; however, several hypotheses have been proposed, including the presence of Howell–Jolly bodies or crystallized sickle hemoglobin.^41^^,^^42^ Our previous study also suggested that mechanical damage to blood cells during capillary sampling may lead to the aggregation of platelets and fibrin, thereby resulting in an abnormal scattergram.^14^ Abnormal scattergrams occurred in 7.2% (*n* = 89) of our samples, slightly higher than approximately 5% in hospital venous samples without transport (Tougan T et al., unpublished data). The frequency appeared to be lower in the samples collected in Roo (6.0%), Ragwe (4.5%), and Ngeri (3.0%) primary schools, which are closer than the other sites to Homa Bay District Hospital (Figure 1), suggesting that transportation time rather than finger-prick blood collection may account for this variation. This aspect needs further exploration in future studies as one of the important field operational factors. Notably, the occurrence of an abnormal scattergram was similar in PCR-positive and PCR-negative samples, suggesting no association with *Plasmodium* infection.

A recent challenge in *P. falciparum* diagnosis, particularly concerning HRP2-based RDT, is the emergence and spread of parasites with deletions in the *hrp2* and/or *hrp3* genes.^8^ These genes encode the target proteins for RDTs, and their absence has been widely reported in malaria-endemic regions, leading to false-negative RDT results. We have verified that the XN-31, similar to microscopy, can detect these parasites and address the limitations of RDTs. This concept was previously demonstrated in an in vitro study,^16^ and our study confirmed its validity in a clinical setting. The prevalence of *hrp2/3*-deleted parasites is reportedly increasing,^43^ and we believe that the ability to detect these parasites is critical for future malaria diagnostic strategies.

This study has several limitations. First, the final number of cases analyzed was 1.4% lower than the planned sample size; however, the resulting loss of statistical power was minimal and unlikely to have materially affected the main results. Additionally, the sample sizes for non-*P. falciparum* infections and *hrp2/3*-deleted *P. falciparum* infections were small, rendering the results primarily descriptive. In this study, non-*P. falciparum* infections were analyzed as a single “others” category, which included *P. malariae* and *P. ovale*, and potential species-specific effects on XN-31 performance could not be evaluated because of limited sample sizes. Further assessments of the capability of the XN-31 to detect low-density non-*P. falciparum* infections and *P. falciparum* with *hrp2/3* deletions may provide additional support for its utility in clinical and research settings. In addition, as discussed above, the performance of RDTs warrants careful interpretation. Although we do not have a definitive explanation for the observed relatively high sensitivity and low specificity, this issue does not affect the evaluation of the XN-31, which is the primary focus of this study.

## CONCLUSION

Overall, the XN-31 exhibited slightly better sensitivity and similar specificity compared with microscopy, effectively detected HRP2-deleted parasites, and performed well in afebrile low-density cases. It requires minimal training and provides rapid results. These advantages may offset some of the limitations of the XN-31, such as cost, maintenance, and infrastructure requirements, particularly in contexts where microscopists have diminishing opportunities to observe parasites, thereby hindering skill retention, or in settings where mass testing and treatment are routinely required, such as large-scale interventions toward malaria elimination.

## Supplemental Materials

10.4269/ajtmh.26-0027 Supplemental Materials
