# Supplementary material for: Diagnostic Performance of the Hematology Analyzer XN-31 for Detecting Plasmodium Infections in School-Based Surveillance in Western Kenya
Source: Am J Trop Med Hyg. 2026 Nov 12:tpmd260027. Online ahead of print. doi: 10.4269/ajtmh.26-0027 (PMC13770342; doi:10.4269/ajtmh.26-0027)
Supplement: Supplementary file 1 [file tpmd260027.SD1.pdf]

Supplemental Table 1

		Parasreen-Pf		Parascreen-PAN	
		Positive	Negative	Positive	Negative
**Paracheck**	Positive	606	17	184	439
	Negative	5	609	2	612
**Parascreen-PAN**	Positive	184	2		
	Negative	427	624		

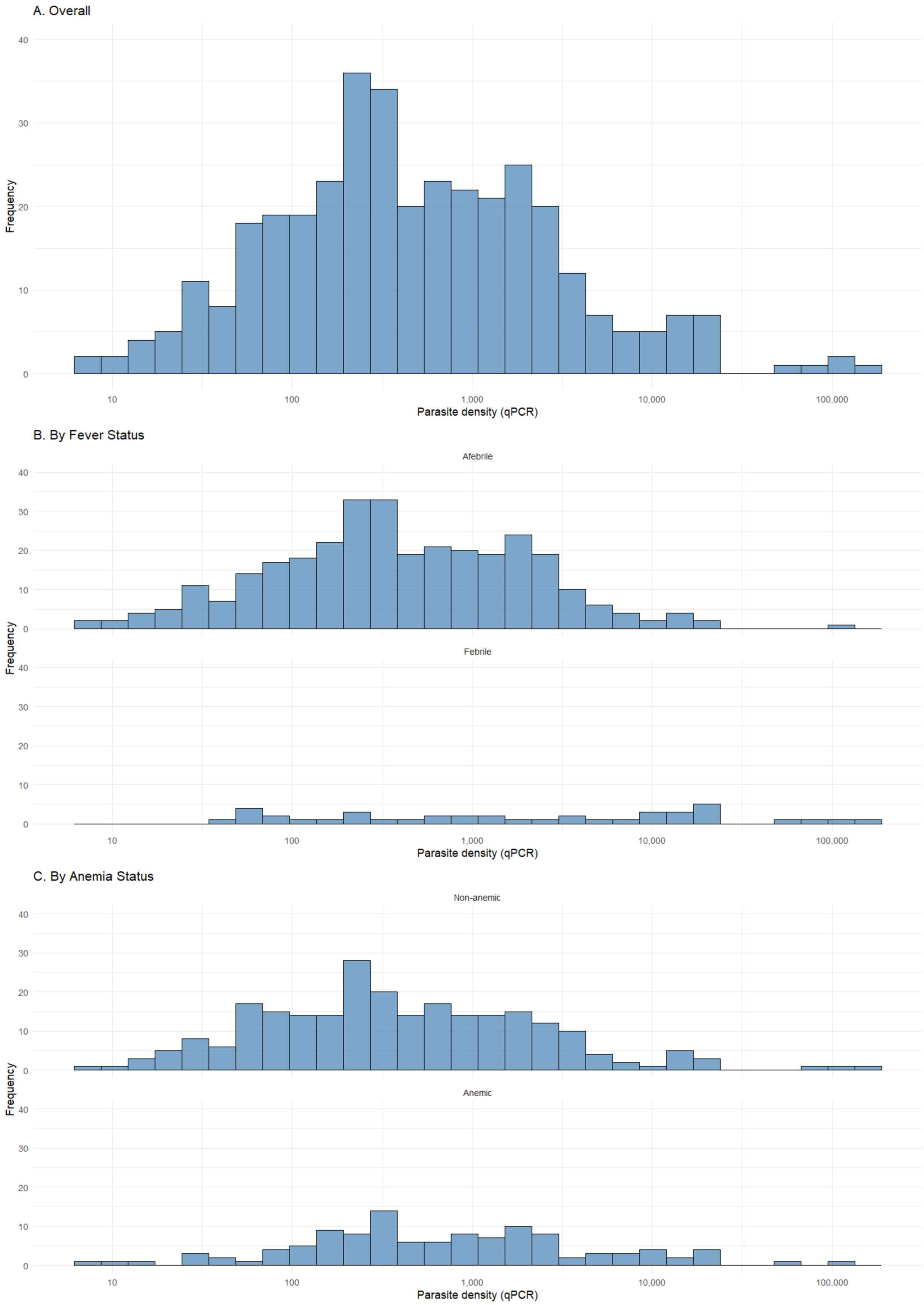

Supplemental Figure 1. Histograms showing the distribution of parasite density (parasite/µL), as measured by qPCR,in (A) the overall study population, (B) afebrile and febrile participants, and (C) non-anemic and anemic participants.
